# Auditing Clinical Outcomes after Introducing Off-Licence Prescribing of Atypical Antipsychotic Melperone for Patients with Treatment Refractory Schizophrenia

**DOI:** 10.1100/2012/512047

**Published:** 2012-04-01

**Authors:** Frank Röhricht, Seema Gadhia, Rinku Alam, Melissa Willis

**Affiliations:** ^1^Newham Centre for Mental Health, East London NHS Foundation Trust and University of Essex, London E13 8SP, UK; ^2^Buckinghamshire NHS Primary Care Trust, Community Pharmacy, Aylesbury HP19 8ET, UK; ^3^North East London NHS Foundation Trust, General Adult Psychiatry, Ilford, Essex IG3 8XJ, UK; ^4^Kent and Medway NHS and Social Care Partnership Trust, Kent ME19 4AX, UK

## Abstract

*Aims and Method*. To evaluate the practical utility of off-licence prescribing and clinical outcomes of treatment with atypical antipsychotic Melperone. *Method*: Prospective data collection on patient's clinical characteristics and outcomes. *Results*. 17 patients with a diagnosis of refractory schizophrenia were identified as suitable for off-license prescribing of Melperone and commenced treatment (13 were previously treated with Clozapine). Seven of those currently remain on Melperone (41%), and for six patents, the BPRS symptom scores reduced significantly over time (24–61%) additionally patients displayed improvements of their quality of life. Six patients were discontinued due to noncompliance and/or side effects. Melperone was ineffective in the other four patients. *Clinical Implications*. The example of a small group of patients responding well to a comparably safe and inexpensive atypical antipsychotic with favourable side effect profile should encourage clinicians to use this tool as third-line treatment and to conduct more systematic clinical research.

## 1. Introduction

The management of chronic, treatment-resistant schizophrenia remains a major challenge for mental health services. The incidence of treatment-resistant schizophrenia has been reported to be between 20% [1] and about one-third of the patients not adequately responding to treatment [2]. With the introduction of the so-called new-generation atypical antipsychotics, greater treatment choice for patients suffering from schizophrenia became available. This is particularly helpful with regard to unpleasant extrapyramidal side effects of the older-generation antipsychotics. Negative symptoms do not respond well to any antipsychotic medication, the most recent UK NICE guidelines for schizophrenia update states that there is currently no conclusive evidence for any antipsychotic having superior efficacy for persistent negative symptoms [3]. 

The only substance with an established evidence base for the treatment of refractory schizophrenia so far is Clozapine. In the key study by Kane et al. [4], 30% of treatment resistant patients responded to Clozapine compared to 4% with Chlorpromazine. The superior efficacy of Clozapine has also been confirmed in a meta-analysis of randomised trials [5]. However, there is a group of patients that do not respond to Clozapine, do not comply with blood-monitoring requirements, or develop serious side effects, which restrict its use. For patients with treatment-resistant, chronic schizophrenia and those who suffer predominantly from negative symptoms, the choice of available treatments (antipsychotics and other) remains therefore rather limited. In Europe, other antipsychotics with favourable side effect profiles, some of them considered to be classified as atypicals, are being used which are unlicensed in the UK (e.g., Perazine, Pipamperone, and Melperone). This group of antipsychotics has not been sufficiently evaluated but may be worth exploring as additional treatment options for patients in the UK and other countries.

Melperone is one such antipsychotic. Despite the fact that it is a butyrophenone derivative, it is said to have atypical properties. It was first used clinically in the 1960s. According to the company's [6] product information, *in vitro* studies show that the affinity of Melperone for the D2 receptor is weaker than for haloperidol and that its antiserotonergic activity (5-HT2 antagonist) is strong. Its anticholinergic and antihistaminergic properties are low. A receptor occupancy study found striatal D2 occupancies of around 70% in patients treated with 250–300 mg Melperone. This level of occupancy is sufficient for clinical effects but too low to raise prolactin levels [7, 8]. It is therefore well tolerated with a low risk of extrapyramidal side effects or tardive dyskinesia at clinically effective doses. Due to its low incidence of extrapyramidal symptoms, Melperone was used in the treatment of iatrogenic psychosis in Parkinson's disease [9]. Melperone is regarded to be a safe antipsychotic drug, and it has been widely used in old-age psychiatry on the continent due to its favourable profile with low antimuscarinergic activity, and a recent study found that Melperone treatment did not result in significant weight gain [10]. There is only one documented case of a fatal intoxication with Melperone [11], the authors describe extremely high concentrations of Melperone in the toxicological analysis and diazepam, and nordazepam and carbamazepine were also detected. 

According to its favourable clinical profile, there has been renewed interest in Melperone following studies suggesting it can be effective in neuroleptic-resistant schizophrenia patients [12, 13]. In September 2005, East London NHS Foundation Trust Medicines Committee approved the use of Melperone for patients with treatment-resistant schizophrenia who have not responded to, or who are intolerant to Clozapine.

## 2. Aim

It is to summarise clinical outcomes for every patient commenced on Melperone in the East London NHS Foundation Trust since approval of its use by the Trust's Medicines Committee. Outcomes will include objective measurement of symptom severity and reasons for stopping treatment with Melperone. Reasons why Clozapine was unsuitable will also be considered.

## 3. Method

The proposal for the prospective study, auditing outcomes (efficacy and safety) following introduction of the new prescribing policy, was submitted to the Trust Audit and Ethics Committee and subsequently approved. Written informed consent was obtained from all participating patients. Clinical notes were searched for the following data.

1. Baseline and subsequent brief psychiatric rating scale/BPRS scores [14]. These should have been documented, as per the guidelines for the use of Melperone (Table 1).
2. Reasons for stopping Clozapine.
3. Maximum dose of Melperone reached.
4. Reason(s) for stopping Melperone.
5. Any additional antipsychotic(s) in use whilst taking Melperone.

Consultant psychiatrists were asked to provide statements regarding clinical observations of change following treatment with Melperone.

## 4. Results

17 patients (6 females and 11 males, different ethnicity, and mean age 39.3 years/sd 10.5, mean number of previous hospitalisations 3.7/sd 2.0, mean duration of illness 13.6 years/sd 7.4) have been started on Melperone since 2005. All included patients had a diagnosis of refractory (treatment resistant) schizophrenia with ongoing both positive and negative symptoms despite exposure to different first- and second-generation antipsychotics. Of these patients, 7 currently remain on Melperone (41%) with a mean daily dose of 340 mg (range 150–600 mg/SD 112). 

Demographic and clinical data for these patients is summarised in Table 2.

We excluded three cases from the analysis for the following reasons: two patients had no BPRS scores measured at all, despite the requirements of the operational policy, and the other two patients had a very low baseline BPRS (<30), questioning the necessity for antipsychotic medication and the criteria for refractory (treatment resistant) schizophrenia; all these four patients did not take Melperone for longer than 2–4 weeks.

### 4.1. Reasons Why Clozapine Treatment Ended

13 of the 17 patients had been prescribed Clozapine previously. One patient had not been prescribed Clozapine previously due to physical health limitations (severe metabolic syndrome/insufficiently controlled diabetes), one due to severe obesity and the other two patients refused to comply with necessary blood monitoring.

Of those 13 patients prescribed Clozapine, it was necessary to end treatment for the following reasons: 3 developed neutropenia, 5 did not respond to treatment, 2 developed myocardiopathy, 1 developed paralytic ileus and severe hypotension, and 2 patients developed severe weight gain/obesity.

### 4.2. Reasons Why Melperone Treatment Was Not Commenced or Ended

Those 10 patients (59%) who did not start or continue treatment with Melperone did so for the following reasons: six patients were non- or insufficiently compliant with the treatment from the beginning. Reason for noncompliance was not given for three of these patients. Headache was cited as the cause for noncompliance in one patient, one patient complained about stomach upset, and one patient said he/she preferred depot injections to taking tablets. 

It was reported that Melperone was ineffective in the other 4 patients whose treatment with Melperone ceased, two of those patients were treated for less than 4 weeks and with a maximum dose of 300 mg; Table 3 summarises their scores. 

Testing the difference between the BPRS total scores at baseline with those after treatment with Melperone (*N* = 7; nonparametric data analysis due to small sample size) the following significant changes were found (Wilcoxon's matched-pairs test): (1) from baseline to 3–6 months treatment: *z* = −2.0, *P* = .043; (2) from baseline to 12–24 months treatment: *z* = −2.2, *P* = .028.

Comments from consultant psychiatrists were recorded for four different patients: (1) (quoting the patient's account) “My head is clearer. I can think more easily”; (2) (quoting a carer's account) “Despite continuing symptoms, the Melperone has made a dramatic difference to my wife's quality of life” and (quoting the care-coordinator's account) “Regardless of previous medications, she had been withdrawn, only poorly attending to her personal hygiene…which now means she can function to a degree and take care of herself and her children with some support”; (3) “Excellent response and went into remission” and “Everyone, even his family said he was a new person and all the staff on the ward were delighted…before he was bouncing back and forth from PICU and hospital for preceding three years”; (4) “Initially he made favourable response with remission of psychotic symptoms apart from transient fleeting paranoid ideas such that we were able to discharge him into the community.”

## 5. Discussion

Since Melperone was introduced as an off-licence prescribed substance into the portfolio of antipsychotics available to patients under the care of East London NHS Foundation Trust (ELFT), only a relatively small number of patients received this treatment over a period of five years. It is important to mention that most patients were started on Melperone by psychiatrists, who previously gained clinical experiences prescribing Melperone in other European countries, where it has been available as a licensed antipsychotic for long time. Only two UK trained psychiatrists included Melperone into their portfolio of prescribing. It appears that despite the fact of a widely acknowledged problem with treatment-resistant schizophrenia and even though a relevant percentage of patients cannot be commenced on or continue treatment with Clozapine, the alternative of off-licence prescribing is not considered suitable by most clinicians. One may speculate that this is due to systemic factors such as risk averse culture in the NHS or concerns regarding the logistics of prescribing, dispensing, and monitoring the drug, but it could also be a result of a therapeutic nihilism and lack of information gathering and circulating.

Most clinical trials evaluating the efficacy of antipsychotic medication define a response as a 20% reduction in BPRS total score. This patient group has had an exhaustible array of treatment options prior to Melperone. We have seen a >20% reduction in six patients, some of them displaying a very significant clinical response, characterised as a “remission” by their clinical teams for two patients. Amongst those four patients not responding to treatment, two were not treated over a sufficient period of time and without increasing the dose to the maximum level. From a clinical perspective, any success in terms of symptom reduction is remarkable in a group of patients with treatment refractory chronic schizophrenia. Following failure of Clozapine exposure, nearly all of the patients in this sample had hardly any pharmaceutical alternative for treatment left. When considering outcome, it is important not to focus on objective measures of psychopathological symptom reduction alone. There has been a clear subjective improvement in all but one of the patients currently taking Melperone. Future work should aim to address such changes in social functioning and subjective quality of life in the context of systematic assessments, using a range of established and validated outcome measures.

Despite the fact that some patients reported mild side effects (mainly tiredness, dizziness, and blurred vision), Melperone was well tolerated in this sample with no occurrence of extrapyramidal symptoms despite relatively high dosages administered. Although doses of 100–300 mg a day are considered effective, doses of up to 600 mg a day are used in Scandinavia, where necessary. A study evaluating the efficacy of two different dosages of Melperone (100 or 400 mg daily) in a sample of 34 acutely hospitalised patients found that both dosages resulted in “ameliorating psychopathology and improving overall psychiatric status” [15]. 

The observed findings can only be discussed with caution; there are relevant methodological limitations. Our sample size of seventeen is small, and given the naturalistic design, the BPRS scores were obtained by doctors currently involved in the patient's care. They were therefore not blind to previous scores. We continue to collect data. It is however clearly encouraging that a relevant proportion of the patients treated with Melperone in this trust have had good clinical outcomes. It is also useful to know that subjective measures (including carer views) could reveal further benefits. Considering the clinical and research implications of this study, it appears that systematic research regarding efficacy and effectiveness, ideally more randomized controlled trials, is needed. However, Melperone is an “off-patent” drug and with no potential for profit; hence, clinical trials conducted by the pharmaceutical industry and/or applications for licensing from pharmaceutical companies will not be forthcoming. Therefore, independent research trials should be conducted by academic institutions with the support of government research grants. In the interest of those patients who do not respond to available treatments and who therefore continue to suffer from refractory symptoms, other Mental Health trusts in the UK should be encouraged to introduce off-license prescribing of Melperone and other antipsychotics with established clinical track record in other countries. Given the experiences so far in this Trust, those initiatives must be accompanied by systematic promoting strategies in order to provide clinicians proactively with necessary information. At the moment, the best possible care for UK patients remains compromised as some treatment options continue to fall by the wayside.

## Figures and Tables

**Table 1:** Guidelines and requirements for the use of Melperone as defined within ELFT medicines committee policy.

Melperone should be prescribed by a consultant psychiatrist.	
The patient's consent for use of an unlicensed drug must be sought and clearly documented in their medical notes.	
The drug is considered for those with treatment-resistant schizophrenia who have not responded to, or cannot tolerate Clozapine.	
Start at 25 mg nocte and increase according to tolerability. In nonrefractory illness, 100–300 mg/day may be effective. Higher doses in refractory illness.	
Full blood count, ECG, and blood pressure prior to commencement.	
Melperone should not be used in conjunction with another antipsychotic.	
Melperone initiation form must be completed to allow monitoring and audit of patients.	
Baseline and subsequent BPRS to allow formal assessments of mental state.	
Prescribing continued by secondary care services.	

**Table 2:** Those 7 patients who are currently continuing with Melperone.

Age	Sex	Ethnicity	Duration illness (years)/prev. hosp.	current dose	Baseline BPRS	Followup BPRS-1 (3–6 months since baseline test)	Followup BPRS-2 (12–24 months since baseline test)	% change between baseline and BPRS-2
54	M	White Cauc.	22/2	450 mg	46	39	35	23.9%
35	F	Asian	18/3	600 mg	58	59	35	32.8%
31	M	Asian	8/4	300 mg	92	63	44	52.2%
32	M	Afro-Carib.	14/4	600 mg	66	72	72	−9.1%
34	M	Black African	5/10	500 mg	67	26	26	61.1%
24	M	Asian	10/5	500 mg	98	45	45	54.1%
36	F	Afro-Carib.	7/2	450 mg	54	28	28	48.2%

**Table 3:** Those 4 patients who stopped Melperone as it is considered “ineffective.”

Age	Sex	Race	Duration illness/number of previous hospitalisations	Duration of treatment	Max. dose reached	Baseline BPRS	Followup BPRS (time since baseline test)
51	M	Asian	20/3	13 months	400 mg	62	63 (6 months)
35	M	Black African	15/4	4 months	600 mg	48	54 (7 weeks)
51	M	White Cauc.	25/5	<1 month	200 mg	Not assessed	Not assessed
49	F	Asian	10/3	<1 month	300 mg	78	75 (3 weeks)
